# Does Chloroplast Size Influence Photosynthetic Nitrogen Use Efficiency?

**DOI:** 10.1371/journal.pone.0062036

**Published:** 2013-04-19

**Authors:** Yong Li, Binbin Ren, Lei Ding, Qirong Shen, Shaobing Peng, Shiwei Guo

**Affiliations:** 1 College of Resources and Environmental Sciences, Nanjing Agricultural University, Nanjing, Jiangsu, China; 2 National Key Laboratory of Crop Genetic Improvement, MOA Key Laboratory of Crop Ecophysiology and Farming System in the Middle Reaches of the Yangtze River, College of Plant Science and Technology, Huazhong Agricultural University, Wuhan, Hubei, China; University of Nottingham, United Kingdom

## Abstract

High nitrogen (N) supply frequently results in a decreased photosynthetic N-use efficiency (PNUE), which indicates a less efficient use of accumulated Ribulose-1,5-bisphosphate carboxylase/oxygenase (Rubisco). Chloroplasts are the location of Rubisco and the endpoint of CO_2_ diffusion, and they play a vital important role in photosynthesis. However, the effects of chloroplast development on photosynthesis are poorly explored. In the present study, rice seedlings (*Oryza sativa* L., *cv.* ‘Shanyou 63’, and ‘Yangdao 6’) were grown hydroponically with three different N levels, morphological characteristics, photosynthetic variables and chloroplast size were measured. In Shanyou 63, a negative relationship between chloroplast size and PNUE was observed across three different N levels. Here, plants with larger chloroplasts had a decreased ratio of mesophyll conductance (g_m_) to Rubisco content (g_m_/Rubisco) and a lower Rubisco specific activity. In Yangdao 6, there was no change in chloroplast size and no decline in PNUE or g_m_/Rubisco ratio under high N supply. It is suggested that large chloroplasts under high N supply is correlated with the decreased Rubisco specific activity and PNUE.

## Introduction

The high grain yields of most crops are dependent upon the supply of nitrogen (N) from fertilizers. The increasing cost and high energy requirement of such fertilizer, together with the adverse environmental effects of N pollution have stimulated much research activity that aiming towards enhancing the efficiency of its use. An important variable is the intrinsic N-use efficiency (NUE) in plants. A key component of NUE is the photosynthetic N-use efficiency (PNUE), defined as net photosynthetic rate (*A*) per unit leaf N content. Approximately 75% of N is allocated to chloroplasts [1], [2] and about 27% of this is in Ribulose–1,5–bisphosphate carboxylase/oxygenase (Rubisco) [3], [4], which carries out the primary fixation of CO_2_ in the Benson-Calvin cycle. Thus, Rubisco plays a pivotal role in PNUE as a major repository of N and an enzyme that limits photosynthetic rate under various conditions.

Due to the low concentration of CO_2_ in the atmosphere and the low affinity for CO_2_, the catalytic effectiveness of Rubisco is poor under ambient conditions [4]–[7]. Rubisco may operate significantly below its potential catalytic capacity in C_3_ plants, suggesting that under high N supply or in high N content leaves, there is excess Rubisco protein serving only as a N storage and not contributing to photosynthesis [8]–[12], especially under limiting light. The lower relative Rubisco activity in high N content leaves may thus contribute to a decreased PNUE in such leaves.

In full sunlight, photosynthesis in C_3_ plants is mainly limited by Rubisco activity [11], [13], [14]. Rubisco activity is related to CO_2_ concentration in chloroplasts [15], and therefore it has been suggested that the decreased Rubisco activity in high N content leaves is due to an insufficient supply of CO_2_ [16]. In the diffusion pathway from atmosphere to chloroplasts, CO_2_ diffuses across a boundary layer above the leaf surface, and then through the stomata into the substomatal cavity. In the substomatal cavity, CO_2_ dissolves in the water-filled pores of the cell wall and then diffuses through the cell wall, the plasma membrane, the cytosol, and the chloroplast envelope to enter the chloroplast. The rate of CO_2_ diffusion from the intercellular spaces to the carboxylation sites in chloroplasts is referred to as the mesophyll conductance, g_m_. It has been demonstrated that g_m_ markedly limits chloroplast CO_2_ concentration relative to intercellular CO_2_ concentration (C_i_) [17]–[20].

It is thought that chloroplast size would probably affect g_m_ [21]. The conductance in the liquid phase in mesophyll cells is the dominant component of g_m_ [17], [22], especially the conductance through the inner chloroplast envelope membrane, which constitutes about one half of total internal resistance [23]. Thus, g_m_ depends upon the conductance per unit of chloroplast surface area and the surface area of chloroplasts facing the intercellular air spaces [22]. Larger chloroplasts are usually correlated with higher N content [24] and would potentially increase g_m_ [25]. A larger chloroplast would also store more leaf N and Rubisco. However, it is not clear whether the extent of the increase in g_m_ is sufficient to provide enough CO_2_ for activating the increased amount of Rubisco, and thus whether an imbalance between the increases in g_m_ and in Rubisco content contributes to the decrease in PNUE observed in high N leaves.

Few studies have specifically investigated the relationship between chloroplast ultrastructure and PNUE that aiming at testing whether larger chloroplasts are related to lowered Rubisco activity and PNUE. We have studied the responses of two rice varieties that respond differently to N supply and provide evidence that links changes in chloroplast size with a deficiency in g_m_ that can explain reduced PNUE and Rubisco activity. Hence, we propose a novel explanation for decreased PNUE under high N supply, and suggest an approach to plant breeding to increase N productivity.

## Results

### Growth response to N supply

The response of both Shanyou 63 and Yangdao 6 to the N supply was as predicted (Table 1). Increases in plant biomass were observed at high N in both cases. There was a decrease in root mass ratio (RMR,  =  root biomass /whole plant biomass), and an increase in leaf mass ratio (LMR,  =  leaf biomass /whole plant biomass) in both varieties with increasing N supply. Leaf sheath and culm mass ratio (SCMR,  =  leaf sheath and culm biomass/whole plant biomass) was unresponsive to N supply, except for a decrease under high N supply in Yangdao 6. SLW was also unresponsive to N supply, indicating no alterations in leaf thickness.

**Table 1:** Growth variables of rice seedlings grown at different N supplies.

Variables	Shanyou 63			Yangdao 6		
	Low-N	Int-N	High-N	Low-N	Int-N	High-N
Plant dry mass	2.92±0.73^b^	4.27±0.60^a^	4.89±0.49^a^	1.80±0.18^c^	2.21±0.45^b^	3.21±.082^a^
(g plant^−1^)						
RMR	0.27±0.02^a^	0.19±0.01^b^	0.12±0.02^c^	0.27±0.02^a^	0.22±0.03^b^	0.15±0.01^c^
(g plant^−1^)						
SCMR	0.48±0.02^a^	0.51±0.02^a^	0.48±0.04^a^	0.47±0.01^a^	0.47±0.04^a^	0.40±0.03^b^
(g plant^−1^)						
LMR	0.26±0.01^c^	0.30±0.02^b^	0.41±0.02^a^	0.26±0.01^c^	0.31±0.02^b^	0.45±0.02^a^
(g plant^−1^)						
leaf area	256±53^c^	444±80^b^	668±44^a^	162±13^c^	235±42^b^	494±116^a^
(cm^2^ plant^−1^)						
SLW	28.98±2.73^a^	29.23±1.15^a^	29.87±2.02^a^	29.19±1.46^a^	29.19±2.06^a^	29.29±1.97^a^
(g m^−2^)						

Rice plants (*cv.* Shanyou 63 and Yangdao 6) were supplied with N at three different levels (low: 20 mg L^−1^ N, intermediate: 40 mg L^−1^ N, and high: 100 mg L^−1^ N). Data are means ± SD of 5 individual plants. Variables were determined 40 days after the start of treatment.

Notes: Significant differences (*P*<5%) between N supplies or varieties were indicated by different lowercase letters or different uppercase letters, respectively. RMR, SCMR and LMR represent root mass ratio, leaf sheath and culm mass ratio and leaf mass ratio, respectively. They were calculated as the ratio of separate dry mass to whole plant dry mass. SLW represents specific leaf weight, and was calculated as the ratio of leaf fresh weight to leaf area.

### Photosynthetic variables

**Figure 1:**
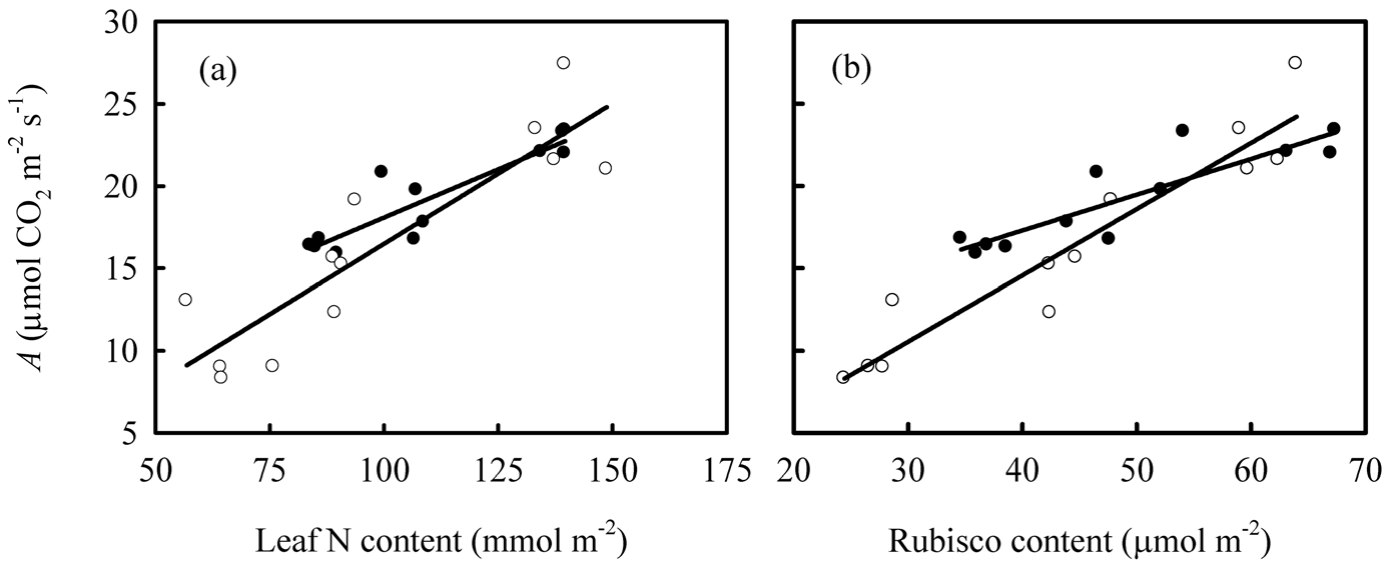
The relationships between leaf photosynthetic rate (*A*) and (a) leaf N content, and (b) Rubisco content and in Shanyou 63 (closed cycles) and Yangdao 6 (open cycles). The lines represent the following regression equations: (a) y = 0.12x+6.33 R^2^ = 0.82 *P*<0.01 for Shanyou 63; y = 0.17x−0.60 R^2^ = 0.79 *P*<0.01 for Yangdao 6; (b) y = 0.22x+8.59R^2^ = 0.78 *P*<0.01 for Shanyou 63; y = 0.40x−1.53 R^2^ = 0.91 *P*<0.01.

In both rice cultivars, *A*, N, NO_3_^−^ and relative Rubisco content were higher under high N supply compared with low N supply (Table 2). However, the responses of these varieties differed markedly when other variables were measured. Most importantly, PNUE (calculated as *A*/N) decreased with increasing N supply in Shanyou 63, but did not change significantly in Yangdao 6. The same trends (decrease in Shanyou 63 and no change in Yangdao 6) were observed in *A*/Rubisco. This phenomenon can also be observed from the relationships between *A* and leaf N content, and relative Rubisco content (Fig. 1). *A*/N and *A*/Rubisco were much lower in high N or Rubisco content leaves in Shanyou 63, with no significant decrease in Yangdao 6 (Fig. 1). Similarly, with increasing N supply, both the initial and maximum Rubisco activities were lower in Shanyou 63, but there were no significant differences in Yangdao 6.

**Table 2:** Effects of N supply level on leaf photosynthesis in rice seedlings.

Variables	Shanyou 63			Yangdao 6		
	Low-N	Int.-N	High-N	Low-N	Int.-N	High-N
*A* (µmol CO_2_ m^−2^ s^−1^)	16.32±0.09^b^	18.76±2.88^ab^	22.68±0.86^a^	10.33±2.32^c^	15.56±2.80^b^	23.36±2.89^a^
g_s_ (mol CO_2_ m^−2^ s^−1^)	0.16±0.03^a^	0.19±0.04^a^	0.18±0.03^a^	0.10±0.04^b^	0.13±0.02^b^	0.21±0.03^a^
g_m__(Harley)_	0.18±0.05^b^	0.21±0.07^ab^	0.25±0.03^a^	0.11±0.03^c^	0.18±0.02^b^	0.26±0.05^a^
(mol CO_2_ m^−2^ s^−1^)						
g_m__(Ethier)_	0.21±0.05^b^	0.24±0.02^ab^	0.28±0.04^a^	0.13±0.01^c^	0.19±0.03^b^	0.28±0.08^a^
(mol CO_2_ m^−2^ s^−1^)						
Г* (µmol CO_2_ mol^−1^)	38.29±0.24^b^	40.80±1.05^ab^	42.26±1.89^a^	38.53±0.68^b^	39.91±1.34^ab^	42.58±1.23^a^
R_d_ (µmol CO_2_ m^−2^ s^−1^)	1.00±0.05^a^	0.67±0.026^b^	0.61±0.06^b^	1.37±0.06^a^	1.23±0.17^a^	0.83±0.09^b^
N (mmol m^−2^)	87.14±0.93^c^	106.43±4.14^b^	143.57±0.50^a^	61.43±4.29^c^	92.14±2.86^b^	139.29±8.57^a^
NO_3_^−^ (mmol m^−2^)	0.20±0.02^b^	0.27±0.07^b^	0.60±0.06^a^	0.20±0.06^b^	0.30±0.07^b^	0.42±0.04^a^
Relative Rubisco content	35.89±2.32^c^	46.79±2.32^b^	61.79±4.29^a^	26.61±2.86^c^	40.89±2.68^b^	60.71±4.11^a^
(µmol m^−2^)						
Chloroplast length	4.20±0.59^b^	4.69±0.62^b^	5.55±0.82^a^	4.51±0.61^a^	4.72±0.63^a^	4.87±0.67^a^
(µm)						
Chloroplast thickness	1.84±0.33^c^	2.34±0.34^b^	3.03±0.49^a^	2.40±0.44^a^	2.30±0.45^a^	2.33±0.46^a^
(µm)						
Initial Rubisco activity	1.05±0.12^a^	0.87±0.04^b^	0.67±0.10^c^	0.69±0.11^a^	0.77±0.21^a^	0.83±0.18^a^
(mol mol^−1^ Rubisco s^−1^)						
Max Rubisco activity	1.41±0.13^a^	1.29±0.13^ab^	1.07±0.23^b^	1.31±0.36^a^	1.38±0.25^a^	1.17±0.06^a^
(mol mol^−1^ Rubisco s^−1^)						
*A*/Rubisco	0.45±0.03^a^	0.40±0.04^ab^	0.36±0.05^b^	0.36±0.06^a^	0.35±0.05^a^	0.38±0.04^a^
(mol CO_2_ mol^−1^ Rubisco s^−1^)						
PNUE (*A*/N)	190±9^a^	178±23^ab^	164±5^b^	154±51^a^	171±27^a^	168±24^a^
(µmol CO_2_ mol^−1^ N s^−1^)						
Chloroplast volume	7.44	13.44	26.67	13.59	13.07	13.84
(V_chl_, µm^3^)						
Chloroplast surface area	18.43	27.33	43.16	27.54	26.82	27.87
(S_chl_, µm^2^)						
g_m_/Rubisco_ (Harley)_	5.02	4.49	4.05	4.13	4.4	4.28
(mol CO_2_ mmol^−1^ Rubisco s^−1^)						
g_m_/Rubisco _(Ethier)_	5.85	5.13	4.53	4.89	4.65	4.61
(mol CO_2_ mmol^−1^ Rubisco s^−1^)						

Rice plants (*cv.* Shanyou 63 and Yangdao 6) were supplied with N at three different levels (low: 20 mg L^−1^ N, intermediate: 40 mg L^−1^ N, and high: 100 mg L^−1^ N). Data are means ± SD of more than 20 individual chloroplasts for their length and thickness, and 5 individual plants for other variables.

Notes: Significant differences (*P*<5%) between N supplies or varieties were indicated by different lowercase letters or different uppercase letters, respectively. *A*, g_s_, g_m_, N, Rubisco and PNUE represent leaf photosynthetic rate, stomatal conductance to CO_2_, mesophyll conductance to CO_2_, leaf nitrogen content, leaf Rubisco content, photosynthetic N-use efficiency, respectively.

**Figure 2:**
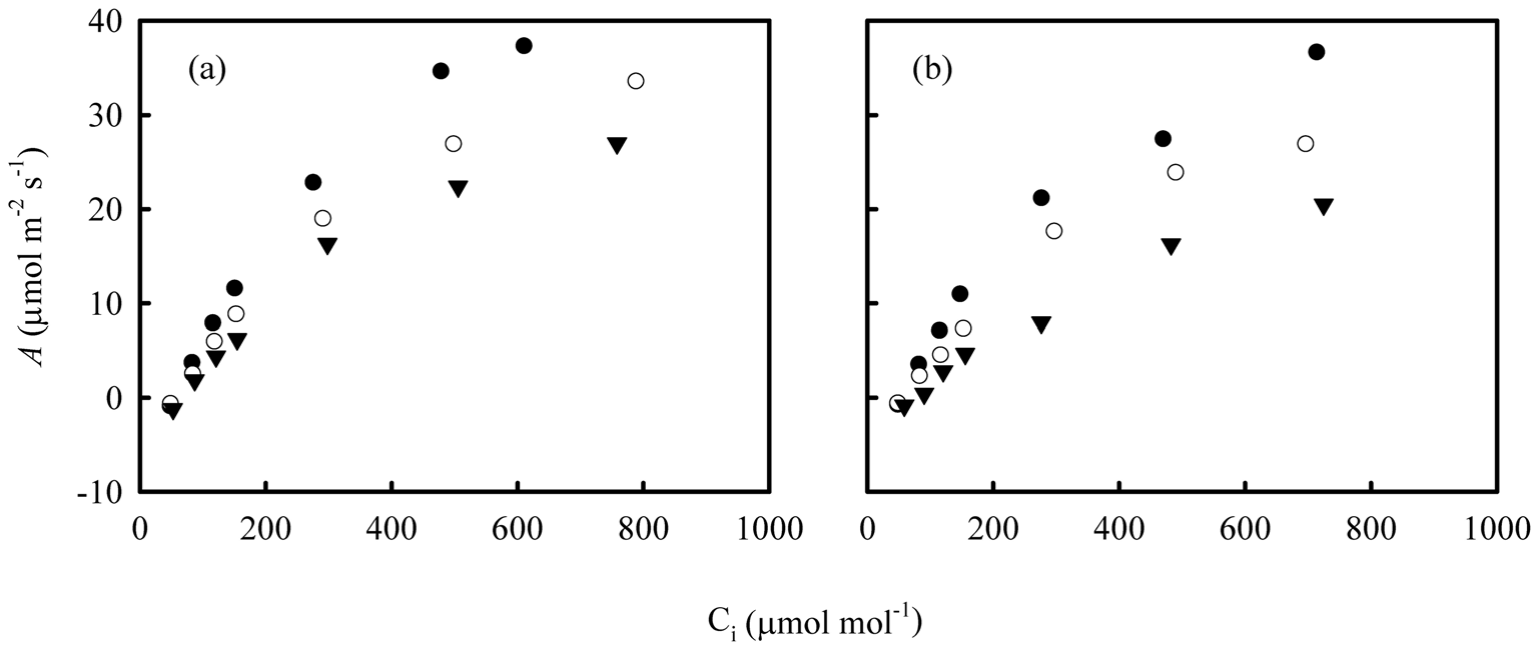
*A*/C_i_ response curves of newly expanded leaves in Shanyou 63 (a) and Yangdao 6 (b). The symbols of solid cycles, open cycles and solid triangles represent high, intermediate and low N supply, respectively.

In Shanyou 63, stomatal conductance (g_s_) was independent of N supply, while in Yangdao 6 it increased at high N supply (Table 2). The values of g_m_ were higher in high N in both varieties. The ratio g_m_/Rubisco declined markedly with increasing N supply in Shanyou 63 but remained constant in Yangdao 6. *A*/C_i_ response curves showed that photosynthesis was more responsive to N supply in Yangdao 6 than in Shanyou 63 (Fig. 2).

### Chloroplast development

**Figure 3:**
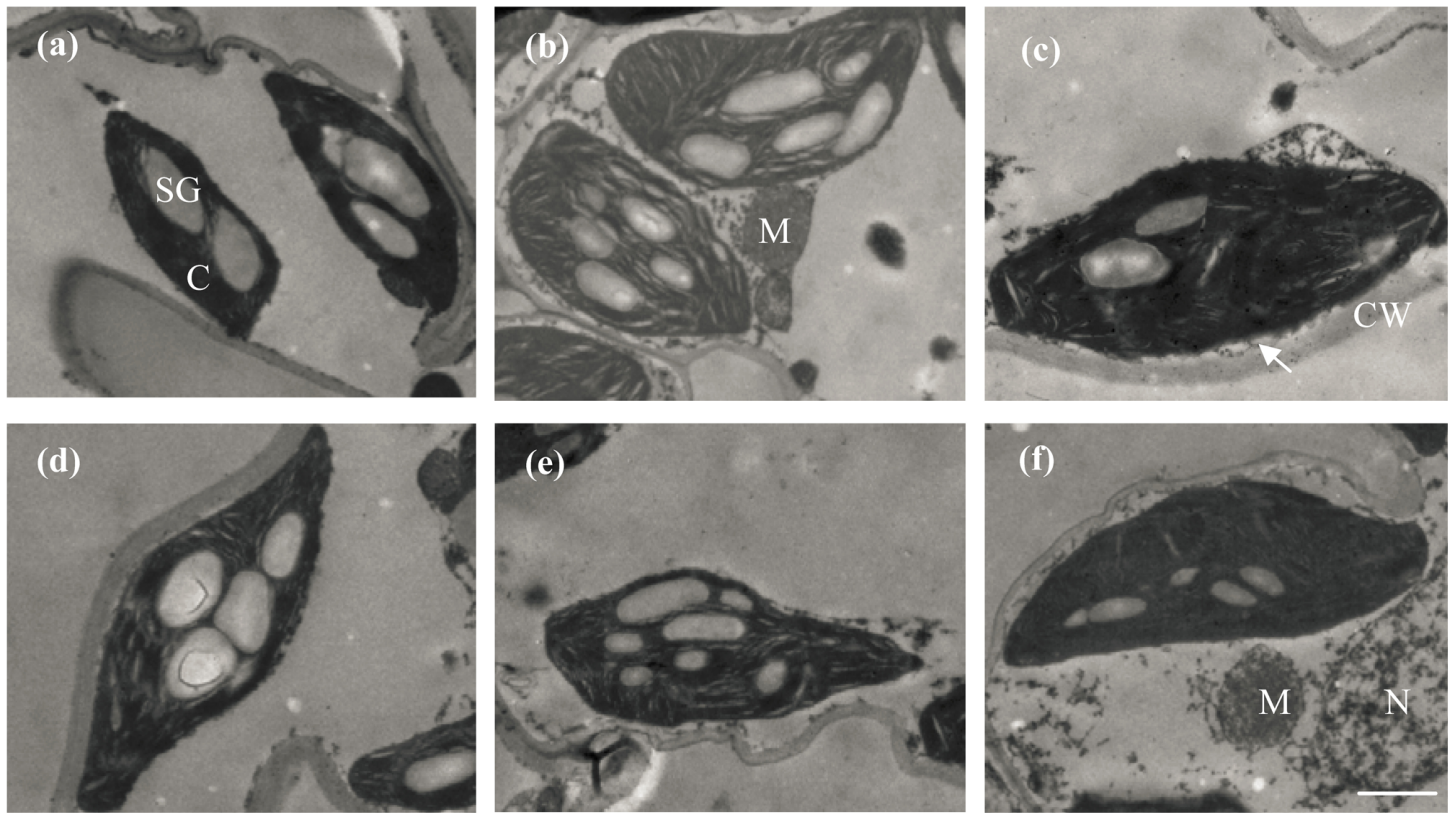
Electron micrographs of chloroplasts in newly expanded leaves of Shanyou 63 (a, low-N supply; b, intermediate.-N supply; c, high-N supply) and Yangdao 6 (d, low-N supply; e, intermediate.-N supply; f, high-N supply). Bar = 1 µm. C, chloroplasts; M, mitochondrion; N, nucleus; SG, starch granules; CW, cell wall; arrows point to plasma membrane.

Chloroplast size also increased with increasing N supply but only in Shanyou 63, with no significant difference observed in Yangdao 6 (Table 2; Fig. 3). Chloroplast length (L_chl_) was less sensitive than chloroplast thickness (D_chl_) to N supply: L_chl_ increased by 32% compared to a 65% increase in D_chl_. Single chloroplast volume (V_chl_) and single chloroplast surface area (S_chl_) increased with increasing N supply in Shanyou 63, but S_chl_/V_chl_ decreased in high N supply. There were no significant differences in V_chl_, S_chl_ and S_chl_/V_chl_ among N supply levels in Yangdao 6 (Table 2). Compared with high N supply, chloroplasts in low N supply showed an accumulation of starch granules in both cultivars (Fig. 3).

## Discussion

### The effect of nitrogen supply on chloroplast development

As much as 75% of leaf N is invested to chloroplasts to synthesis photosynthetic apparatus, including thylakoid membranes and photosynthetic enzymes. Thereby, chloroplast development, such as chloroplast division, chloroplast grana and stroma lamellae stacking, is highly dependent on nitrogen supply. Sufficient N will significantly enlarge chloroplast size, increase chloroplast number, and enhance grana aggregation [26]–[29]. In the present study, chloroplasts were enlarged under high N supply in Shanyou 63, with chloroplast thickness more responsive than chloroplast length (Table 2 and Fig. 3). In contrast, chloroplast size, both chloroplast length and thickness, was insensitive to N supply in Yangdao 6 (Table 2 and Fig. 3).

Under full sunlight, photosynthetic assimilates should be translocated quickly out of chloroplasts to sites with high carbon sink activity to avoid starch granules formation, which will in turn inhibit leaf photosynthesis [28], [30]. It is reported that there are more and larger starch granules under high N supply for their high leaf photosynthetic capacity [27]; however, there are also numerous studies showed less and smaller starch granules under high N supply [26], [28], [30]. In the present study, starch granules were much larger under low N than under high N supply (Fig. 3). The reason is probably that high N supply can stimulate the translocation of assimilates from chloroplasts to sites with high carbon sink activity [26].

### The relationship between Rubisco content and total chloroplast volume

**Figure 4:**
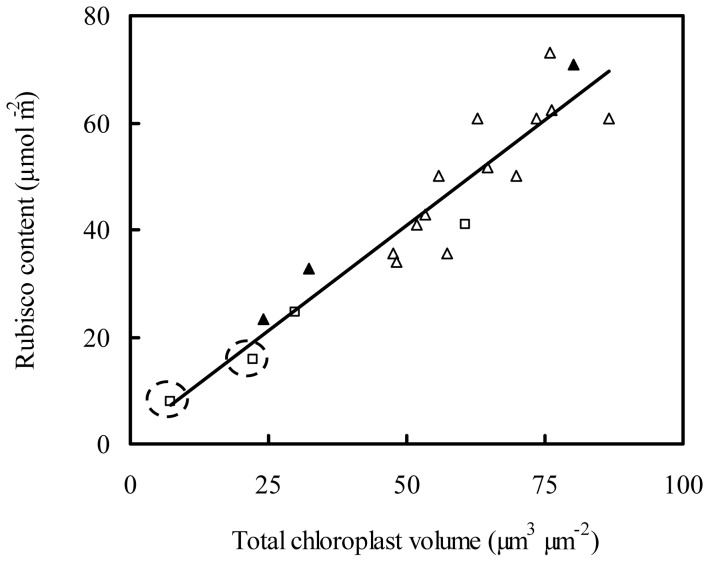
The relationship between Rubisco content and total chloroplast volume per leaf area. The line represents the regression equation: y = 0.79x+1.36, R^2^ = 0.88, *P*<*0.01*. Data sources: *Nicotiana tabacum* □ [21]; *Chenopodium album* ▴ [48]; *Aucuba japonica* Thunb. △ [24]. The two data points in dotted cycles are from transgenic tobacco with a reduced Rubisco content.

Although the level of Rubisco is sometimes excessive for photosynthesis [12], [19], [31], the increase in leaf N content at high N supply is generally accompanied by a higher Rubisco content. In the present study, this was observed in both the rice varieties, Shanyou 63 and Yangdao 6. A higher amount of Rubisco potentially be associated with either a larger total chloroplast volume (V_T–chl_) or a higher Rubisco concentration in chloroplasts (Rub_chl_). Analysis of data from a number of different plant species reveals a linear relationship between Rubisco content and V_T–chl_ expressed on a leaf area basis (Fig. 4). This points to a constant value of Rub_chl_, at approximately 44 mg cm^−3^ (the slope of the regression equation in Fig. 4). Thus, the higher Rubisco content in high N leaves should be associated with a larger V_T–chl_, this hypothesis is also speculated by Evans et al. [32]. The increase in V_T–chl_ could arise from an increase in either V_chl_ or chloroplast number per leaf area (n_chl_).

In Yangdao 6, there was no difference in chloroplast size (Table 2 and Fig. 3), despite the higher Rubisco content in leaves with higher leaf N. In this case, it is suggested that the number of chloroplasts should increase. In contrast, in Shanyou 63, the thickness and the length of chloroplasts were larger in the high N leaves compared to those with low N supply, and the higher Rubisco content at high N supply in Shanyou 63 was hence associated with a larger chloroplast volume. Wider and longer chloroplasts not only have a larger S_chl_, but also a larger V_chl_ and a decreased S_chl_/V_chl_ compared to smaller ones.

### The effect of chloroplast size on mesophyll conductance, g_m_

The total surface area of chloroplasts facing the intercellular space (S_c_) is the key determinant of g_m_, rather than the total chloroplast surface area *per se*. S_c_ is given by S_c_  = α×S_chl_×n_chl_, where α is the ratio of chloroplast surface area facing the intercellular space to total chloroplast surface area. S_c_ is therefore also higher when there are larger chloroplasts, the higher S_c_ with larger chloroplasts would potentially increase g_m_. The increase in S_c_ is again not proportional to the increase in total chloroplast volume, V_T–chl_ ( = V_chl_×n_chl_) and therefore the ratio S_c_/V_T–chl_ (which can be given as (α×S_chl_×n_chl_)/(V_chl_×n_chl_), and further as α×S_chl_/V_chl_) also decreases in large chloroplasts (Table 2). In fact, α is also likely to decrease when chloroplasts are thicker, so amplifying the decrease in this ratio. Hence, it can be concluded that the increase in chloroplast size on Shanyou 63 would be associated with an increase in g_m_, while it would not be proportional to the increase in total chloroplast volume and Rubisco content (Table 2). In contrast, in Yangdao 6, where chloroplast size did not change, the increase in g_m_ at high leaf N would be attributable to an increase in chloroplast number, with no change in the S_c_/V_T–chl_ ( = α×S_chl_/V_chl_) and g_m_/Rubisco ratio (Table 2).

Recent studies demonstrated that photorespiration can efficiently affect the precision of g_m_ estimation [33], [34]. But it is now still difficult for the methods of both simultaneous measurement of gas exchange and chlorophyll fluorescence, and *A*/C_i_ reponse curve-fitting method to exclude photorespiration’s effect. It should be clear that g_m_ in the present study was estimated without eliminating photorespiration effects. In the present study, two independent methods were conducted to improve the accuracy for g_m_ estimation. It should be proposed that, further efforts should be done to improve accuracy for g_m_ estimation in the method of simultaneous measurement of gas exchange and chlorophyll fluorescence to continue its convenience in g_m_ estimation.

It is illustrated that more than 95% of mesophyll cell periphery in rice plants is covered by chloroplasts in well-grown and N sufficient leaves [35]. So, when mesophyll surface is mainly covered by chloroplasts under high N supply, g_m_ and photosynthesis would probably be insensitive to chloroplast development if C_liq_ is similar. This phenomenon was observed in the present study, where photosynthesis and g_m_ were similar between the two cultivars under high N supply while chloroplast size was substantially larger in Shanyou 63 (Table 2). When less mesophyll surface is covered by chloroplasts under low N supply, small chloroplasts would be benefit for g_m_ and photosynthesis because they can more efficiently cover mesophyll cell surface and enhance g_m_ (Table 2).

### Would chloroplast size affect PNUE at high N?

**Figure 5:**
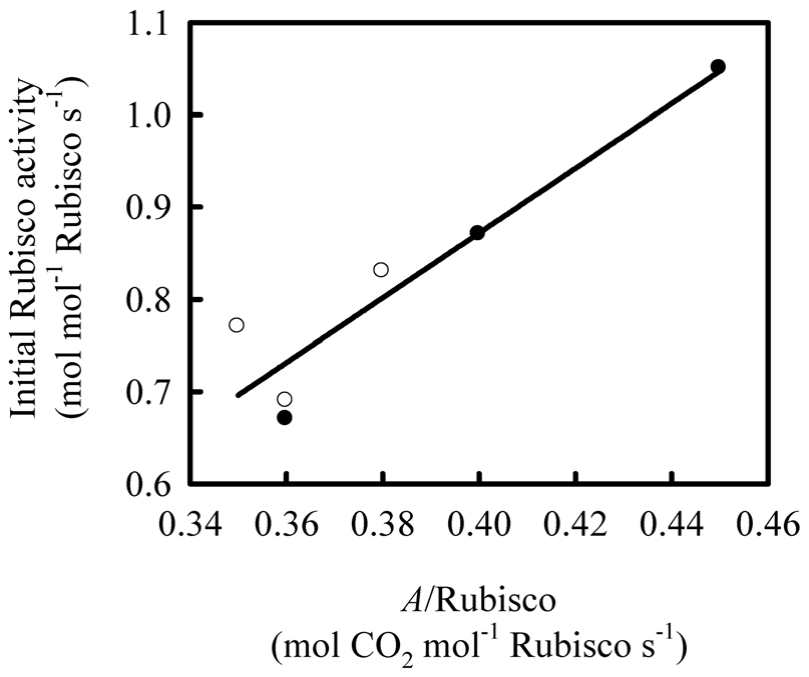
The relationship between initial Rubisco activity and the ratio of leaf photosynthetic rate (*A*) to Rubisco content on Shanyou 63 (solid cycles) and Yangdao 6 (open cycles). The line represents linear regression: y = 3.51x−0.53 R^2^ = 0.88 *P*<0.01.

Rubisco activity can be measured under both *in vivo* and *in vitro* conditions. Because of different synthesis conditions, *in vitro* Rubisco activity can not always reveal its *in vivo* activity especially under drought stress [36]. Nevertheless, there are numerous studies showed the positive relationship between them [31], [37], [38]. The correlation was also checked in the present study (Fig. 5), and the positive relationship revealed that the slowed-down Rubisco turnover rate with increasing N supply in Shanyou 63 was probably the reason for the decreased *A*/Rubisco and PNUE.

**Figure 6:**
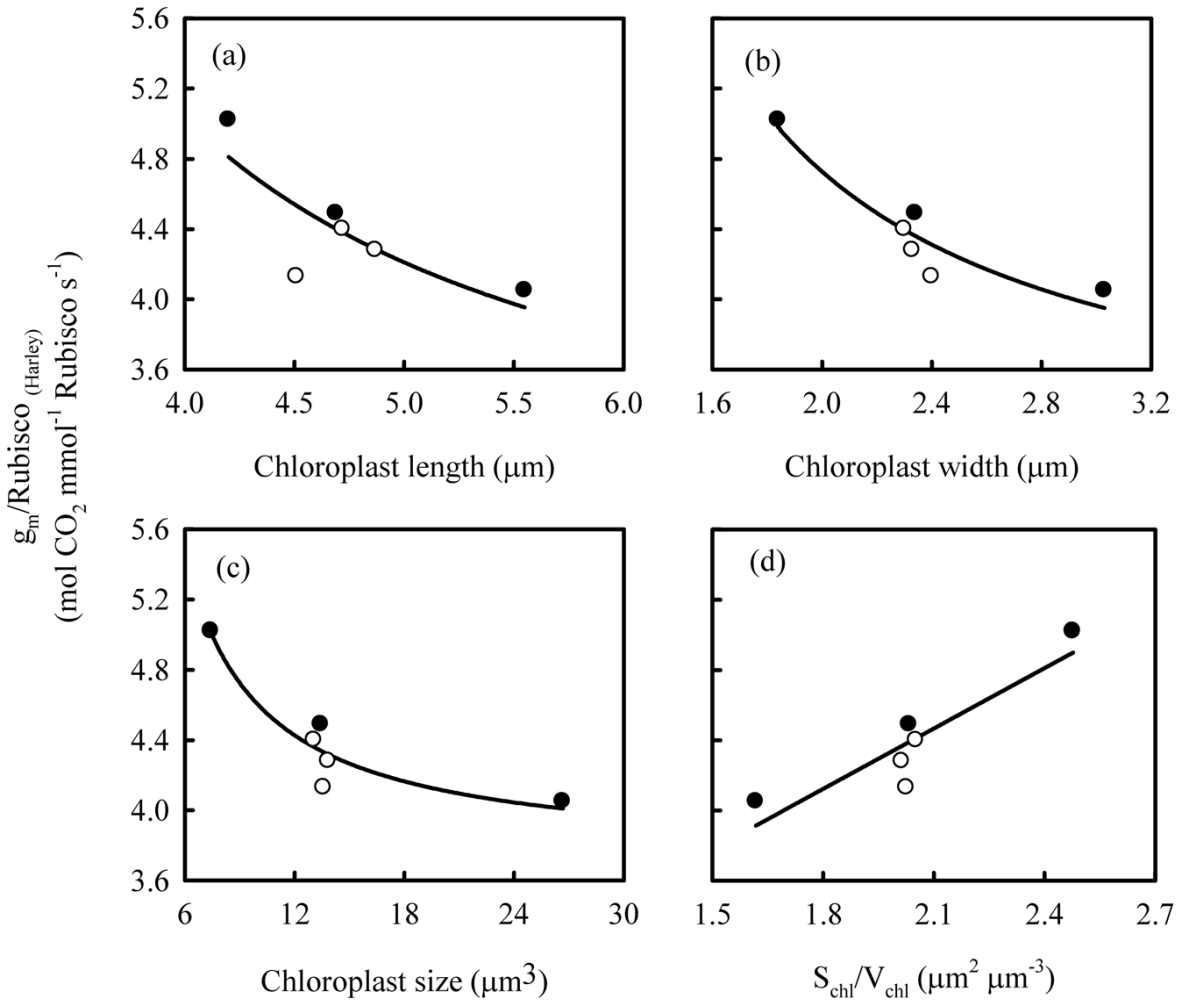
The relationships between chloroplast size and the ratio of mesophyll conductance (g_m_) to Rubisco content on Shanyou 63 (solid cycles) and Yangdao 6 (open cycles). Chloroplast surface area (S_chl_) and volume (V_chl_) were calculated from the Cesaro formula. The lines represent the following regressions: (a) y = 2.54x/(x−1.98) R^2^ = 0.63 *P* > 0.05; (b) y = 2.99x/(x−0.73) R^2^ = 0.88 *P*<0.01; (c) y = 3.72x/(x−1.91) R^2^ = 0.89 *P*<0.01; (d) y = 1.15x+2.06 R^2^ = 0.82 *P*<0.05.

**Figure 7:**
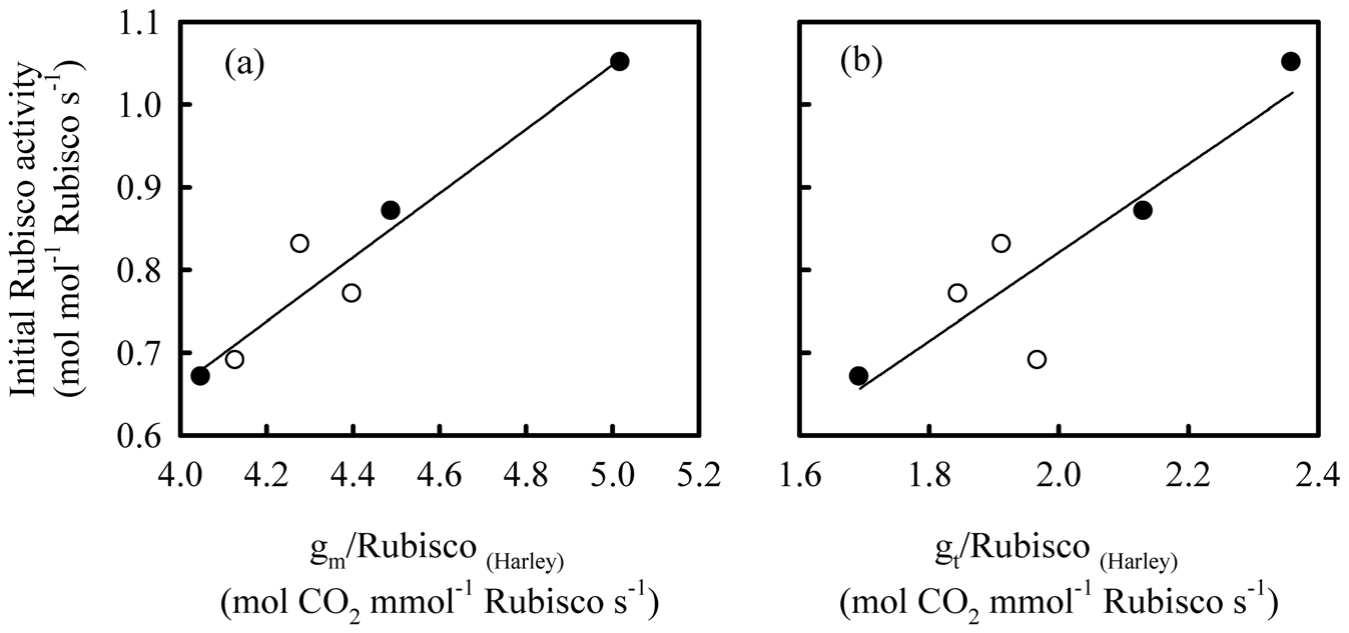
The relationships between initial Rubisco activity and (a) the ratio of mesophyll conductance (g_m_) to Rubisco content, and (b) the ratio of total conductance (g_t_) to Rubisco content on Shanyou 63 (solid cycles) and Yangdao 6 (open cycles). The lines represent the following regressions: (a) y = 0.39x−0.89 R^2^ = 0.93 *P*<0.01; (b) y = 0.54x−0.25 R^2^ = 0.80 *P*<0.05.

**Figure 8:**
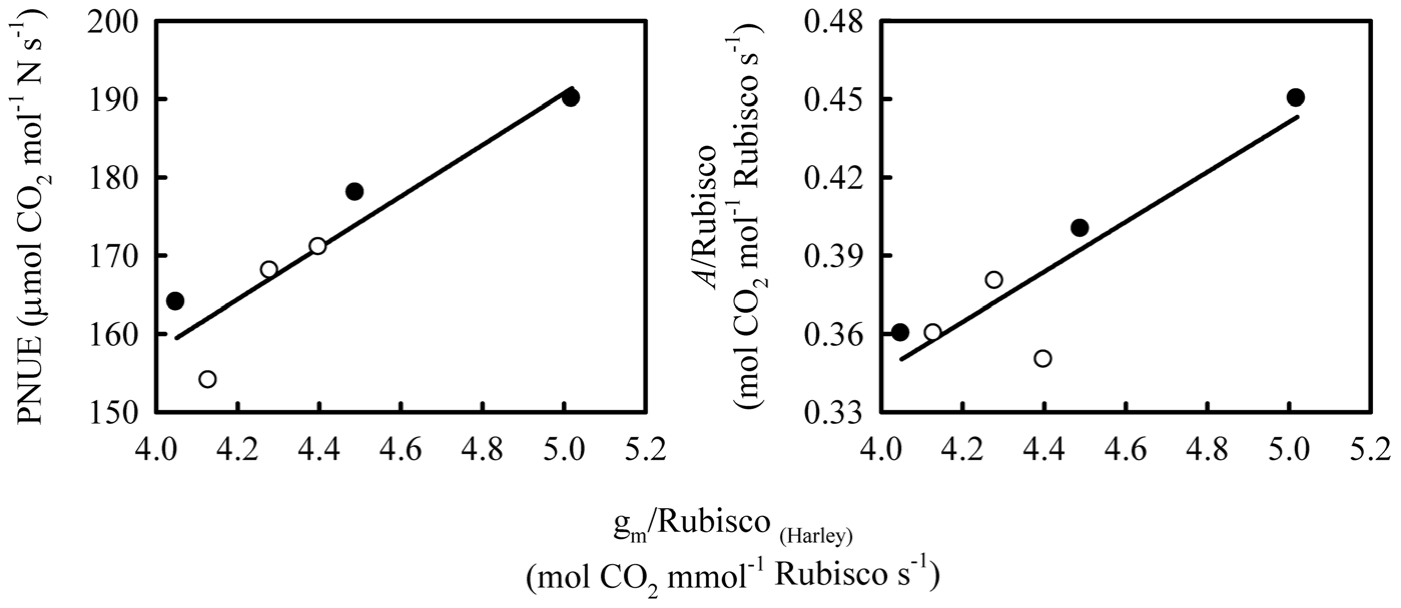
The relationships between the ratio of mesophyll conductance (g_m_) to Rubisco content and (a) photosynthetic N-use efficiency (PNUE) and (b) the ratio of leaf photosynthetic rate (*A*) and Rubisco content on Shanyou 63 (solid cycles) and Yangdao 6 (open cycles). The lines represent the following regressions: (a) y = 32.89x+26.28 R^2^ = 0.86 *P*<0.01; (b) y = 0.096x−0.038 R^2^ = 0.80 *P*<0.05.

In large chloroplasts, the ratio of S_c_ to Rubisco content ( = Rub_chl_×V_T–chl_) is lower, and hence g_m_ per unit Rubisco content also lower, compared to the values in smaller chloroplasts (Fig. 6). Because of the correlation between g_m_ and total conductance (g_t_), responses of g_t_/Rubisco to chloroplast size were similar with those of g_m_/Rubisco (Data not shown). The lower g_m_/Rubisco and g_t_/Rubisco ratio would result in an insufficient supply of CO_2_ in chloroplasts and a consequent decrease in Rubisco activity (Fig. 7). A decrease in g_m_/Rubisco and a reduction in Rubisco activity at high leaf N were observed in Shanyou 63 but not in Yangdao 6 (Table 2). As a consequence, in the former variety, there was a decreased *A*/Rubisco and PNUE, whereas these variables were unchanged in the latter variety. Therefore, the lower g_m_/Rubisco ratio induced by chloroplast enlargement under high N supply in Shanyou 63 at least partially explains the lowered Rubisco efficiency and decline in PNUE (Fig. 8). However, in Yangdao 6, the constant g_m_/Rubisco would ensure a sufficient supply of CO_2_, and thus Rubisco efficiency and PNUE would not decrease under high N supply. It is suggested that whether PNUE decreases under high N would, at least partially, depends on the mechanism by which the Rubisco content increases i.e. whether through increasing V_chl_ or by increasing n_chl_. Thus, we hypothesize that a high N–dependent increase in chloroplast size would cause a decrease in g_m_ per unit Rubisco that results in a fall in PNUE, an effect that does not occur if the response to high N is an increase in chloroplast number.

**Figure 9:**
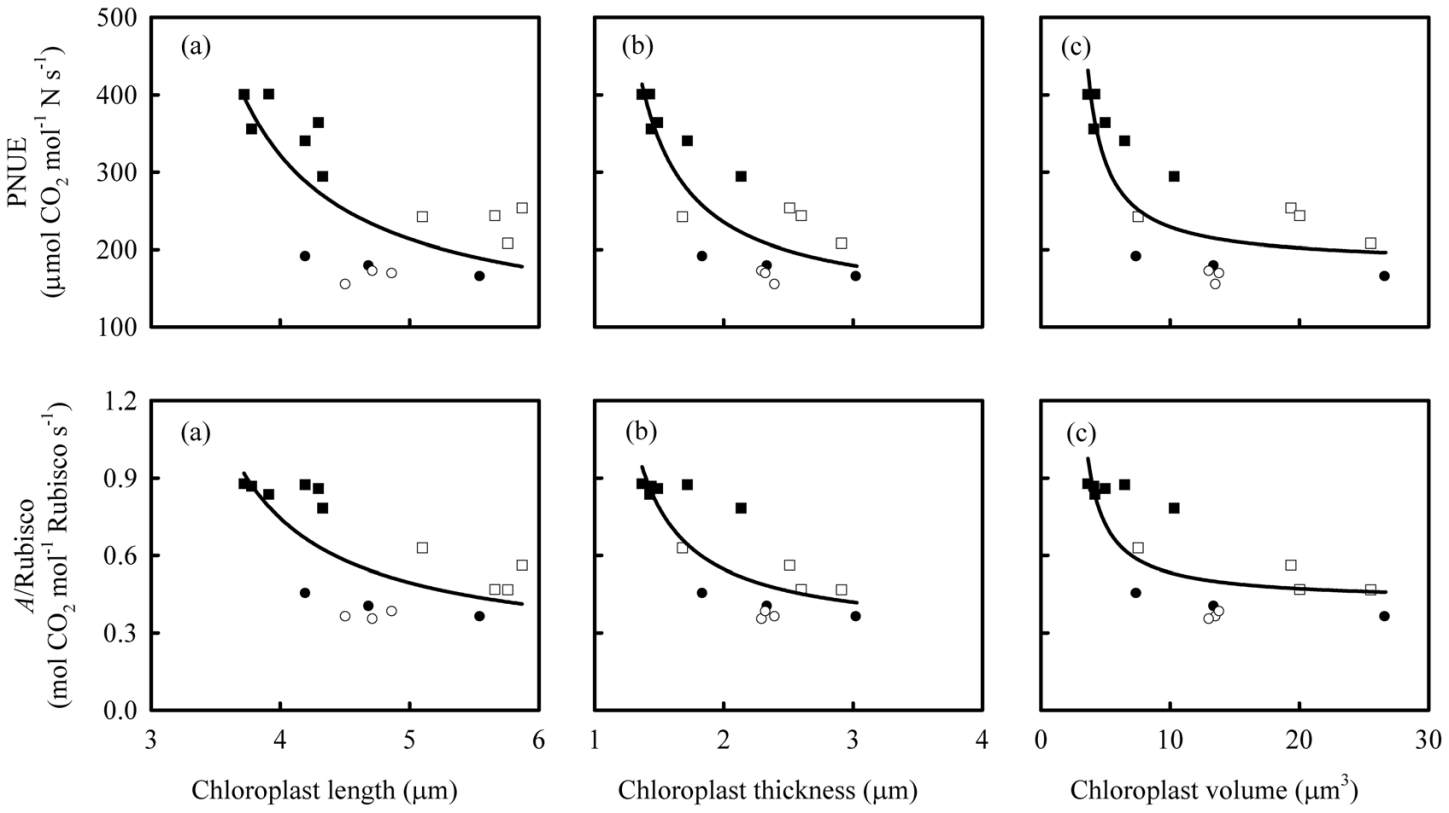
The relationships between chloroplast size and photosynthetic N-use efficiency (PNUE), and the ratio of leaf photosynthetic rate (*A*) to Rubisco. The lines represent the following regression equations: (a) y = 91.04x/(x–2.87), R^2^ = 0.53, *P*<0.01; (b) y = 121.76x/(x–0.96), R^2^ = 0.75, *P*<0.01; (c) y = 180.56x/(x–2.12), R^2^ = 0.72, *P*<0.01; (d) y = 0.21x/(x–2.86), R^2^ = 0.53, *P*<0.01; (e) y = 0.29x/(x–0.95), R^2^ = 0.69, *P*<0.01; (f) y = 0.42x/(x–2.06), R^2^ = 0.63, *P*<0.01. Data sources: data of solid squares were collected from Wuyujing 3 (*Oryza sativa* L. ssp. japonica) with different N supplies; data of open squares were from Shanyou 63 with different N forms and water supply [42]; data of solid and open cycles were from Shanyou 63 and Yangdao 6 with different N supplies.

This hypothesis was tested for its general applicability in rice plants grown under different conditions (Fig. 9). The results showed that PNUE and *A*/Rubisco were negatively related to chloroplast size. The relationships were again stronger with chloroplast thickness than chloroplast length. With similar Rubisco content, leaves with smaller chloroplasts have a higher CO_2_ assimilation rate. Thus, the lack of balance between g_m_ and Rubisco content as chloroplasts increase in size is a tenable explanation of observed decreases in Rubisco turnover rate and the lowered PNUE.

## Materials and Methods

### Plant material and growth conditions

After germination on moist filter paper, rice seeds (*Oryza sativa* L., ssp. indica hybrid, *cv.* ‘Shanyou 63’, and ssp. indica inbred, *cv.* ‘Yangdao 6’) were transferred to 2.0 mM CaSO_4_ for germination at 25±5°C. After 3 days, the rice seedlings were transferred to 6–L rectangular containers (30×20×10 cm) and ¼-strength nutrient solution (for composition, see below). Three days later, the seedlings were transferred to a ½-strength nutrient solution, and after 5 days, the seedlings were supplied with full–strength nutrient solution for 1 week. Seedlings were supplied with nutrient solution containing three different N levels: low N (20 mg L^−1^), intermediate N (40 mg L^−1^), and high N (100 mg L^−1^). The N sources were equimolar amounts of (NH_4_)_2_SO_4_ and Ca(NO_3_)_2_. In addition, the macronutrients in the solution were as follows (mg L^−1^): 10 P as KH_2_PO_4_, 40 K as K_2_SO_4_ and KH_2_PO_4_, and 40 Mg as MgSO_4_. The micronutrients were (mg L^−1^): 2.0 Fe as Fe–EDTA, 0.5 Mn as MnCl_2_·4H_2_O, 0.05 Mo as (NH_4_)_6_Mo_7_O_24_·4H_2_O, 0.2 B as H_3_BO_3_, 0.01 Zn as ZnSO_4_·7H_2_O, 0.01 Cu as CuSO_4_·5H_2_O, and 0.0028 Si as Na_2_SiO_3_·9H_2_O. To compensate for the lower Ca, additional CaCl_2_ was added to the low N and intermediate N treatments. A nitrification inhibitor (dicyandiamide) was added to each nutrient solution to prevent the oxidation of ammonium. Nutrient solutions were changed every 2 days, and the pH was adjusted to 5.50±0.05 every day with HCl or NaOH. All treatments were planted in 5 individual containers and were placed in a completely randomised design.

Plants were grown in a greenhouse at 25/18°C day/night temperature. Light was supplied by SON–T AGRO 400W bulbs, with the light intensity maintained at a minimum of 1000 µmol photons m^−2^ s^−1^ (PAR) at the leaf level and a 14 h photoperiod.

### Gas exchange and fluorescence measurements

Forty days after the start of treatment, photosynthesis and chlorophyll fluorescence were simultaneously measured on light–adapted leaves using a Li–Cor 6400 infrared gas analyzer. Leaf temperature during the measurement was maintained at 30.5±1.1°C, with a photosynthetic photon flux density (PPFD) of 1500 µmol photons m^−2^ s^−1^. The CO_2_ concentration in the cuvette was adjusted to the ambient CO_2_ concentration (424.3±1.9 µmol mol^−1^), and the relative humidity was maintained at 50%. After equilibration to a steady state, the fluorescence was measured (F_s_) and a 0.8 s saturating pulse of light (approx. 8000 µmol m^−2^ s^−1^) was applied to measure the maximum fluorescence (F_m_’). Gas exchange variables were also recorded simultaneously. The efficiency of photosystem II (Φ_PSII_) was calculated as Φ_PSII_  = 1−F_s_/F_m_’.

Total electron transport rate (J_T_) was calculated as J_T_ = Φ_PSII_×PPFD×α_leaf_×β, where α_leaf_ and β were leaf absorption and the proportion of quanta absorbed by photosystem II, respectively. The product α_leaf_×β was determined from the slope of relationship between Φ_PSII_ and the quantum efficiency of CO_2_ uptake (Φ_CO2_), obtained by varying light intensity under non–photorespiratory conditions at less than 2% O_2_ [39]. The variable J_T_ method [17] was used to calculate g_m_ using the equation g_m_ = *A*/{C_i_−Γ*[J_T_+8(A+R_d_)]/[J_T_−4(A+R_d_)]}, where *A* is the rate of leaf photosynthetic CO_2_ uptake per unit leaf area, Г* is CO_2_ compensation point and R_d_ is the rate of dark respiration. Г* and R_d_ were measured following Laisk’s method [40], as described by Brooks and Farquhar [41] with minor modifications. The process was described in detail in our previous study [42]. g_t_ was calculated as g_t_ = g_s_×g_m_/(g_s_+g_m_) [43].

After the above gas exchange measurement, *A*/C_i_ response curves were conducted on the same leaves. Leaf temperature, PPFD, and relative humidity during measurements were maintained as mentioned above. Prior to measurements, leaves were placed in the cuvette at a PPFD of 1500 µmol photons m^−2^ s^−1^; CO_2_ concentration in the cuvette was maintained at 400 µmol CO_2_mol^−1^ with a CO_2_ mixer. Ten minutes later, CO_2_ concentration in the cuvette was controlled across a series of 1000, 800, 600, 400, 200, 150, 100, and 50 µmol CO_2_ mol^−1^. After equilibration to a steady state, data were recorded automatically. g_m_ was then calculated based on gas exchange measurements themselves according to the method in Ethier and Livingston [44], which is modified from that in Farquhar et al. [13].

### Relative Rubisco content and activity measurements

The Rubisco content of newly expanded leaves was determined according to the method of Makino et al. [45], [46]. Briefly, samples of newly expanded leaves were immersed in liquid N, and then stored at −70°C. For analysis, 0.5 g were ground in a solution containing 50 mM Tris–HCl (pH 8.0), 5 mM β–mercaptoethanol, and 12.5% glycerol (v/v), and then centrifuged at 1500 g for 15 min at 4°C. The supernatants were mixed with a solution containing 2% (w/v) SDS, 4% (v/v) β–mercaptoethanol and 10% (v/v) glycerol, boiled in a water bath for 5 min before SDS–PAGE using a 4% (w/v) stacking gel, and a 12.5% (w/v) separating gel. After electrophoresis, the gels were stained with 0.25% Commassie Blue for 12 h, and destained. Gel slices containing the large subunits and small subunits of Rubisco were transferred to a 10-mL cuvette containing 2 ml of formamide and incubated at 50°C in a water bath for 8 h. The absorbance of the wash solution was measured at 595 nm. Protein concentrations were determined using bovine serum albumin as a standard.

Rubisco activity was measured according to Jin et al. [7] with minor modification. Briefly, about 0.1 g of newly expanded leaves were ground with 2 ml of a solution containing 50 mM Tris–HCl (pH 7.5), 10 mM β–mercaptoethanol, 12.5% (v/v) glycerol, 1 mM EDTA–Na_2_, 10 mM MgCl_2_, and 1% (m/v) PVP–40. After centrifugation at 15,000 *g* for 1 min at 4°C, the activity in the supernatants were assayed. The initial Rubisco specific activity was measured at 30°C by adding 100 µL of the supernatant to 900 µL of assay solution containing 56 mM HEPES–NaOH (pH 7.5), 1 mM EDTA–Na_2_, 20 mM MgCl_2_, 3 mM DTT, 11 mM NaHCO_3_, 6 mM ATP, 6 mM creatine phosphate, 0.2 mM NADH, 11 units of phosphocreatine kinase, 11 units of glyceraldehyde–3–phosphate dehydrogenase, 11 units of phosphoglycerate kinase, 11 mM Tris–HCl (pH 7.5), and 0.7 mM RuBP. The absorbance at 340 nm was recorded at 3 s intervals for 30 s. To measure total Rubisco specific activity, 100 µL of the supernatant were added to 200 µL of activation medium containing 1 mM EDTA–Na_2_, 50 mM MgCl_2_, 15 mM NaHCO_3_, and 50 mM Tris–HCl (pH 7.5), and incubated at 30°C for 10 min. Following the addition 700 µL of assay solution, the activity was determined by recording the absorbance at 340 nm at 3 s intervals for 30 s.

### Chloroplast ultrastructure

Leaf pieces of approximately 1–2 mm^2^ were cut from the middle of newly expanded leaves using two razor blades, then they were fixed in 2.5% glutaraldehyde in 0.1 M phosphate buffer, pH 7.4, and post–fixed with 2% osmium tetroxide. Specimens were dehydrated in a graded acetone series and embedded in Epon 812. Leaf sections, 70 nm thick, were cut with a Power Tome–XL ultramicrotome, stained with 2% uranyl acetate, and examined under an H–7650 transmission. Chloroplast length and thickness were calculated from at least 20–30 chloroplasts. Chloroplasts were assumed to be ellipsoids of revolution, which is a shape that is generated by rotating an ellipse around one of its axes. According to the Cesaro formula [47], S_chl_ was calculated as S_chl_  = 4×π×(a×b^2^)^2/3^, where a =  L_chl_/2, b =  D_chl_/2. V_chl_ was calculated from the Cesaro formula, V_chl_  = (4/3)×π×a×b^2^.

### Measurement of biomass, leaf nitrate and N content

Nitrate from newly expanded leaves was extracted in boiling water for 30 min and reacted with salicylic acid, and the color was determined at a wavelength of 410 nm. 45 days after treatments were started when all measurements had been completed, plants were harvested and separated into root, leaf sheath and culm, and leaf fractions. Leaf area and leaf fresh weight were determined and specific leaf weight (SLW) was calculated as the ratio of leaf fresh weight to leaf area. All samples were oven-dried at 105°C first for 30 min, and then at 70°C to constant weight. The dried leaves were digested with H_2_SO_4_–H_2_O_2_ at 260–270°C, and the total leaf N concentration was determined using a digital colorimeter (AutoAnalyzer 3; Bran+Luebbe).

### Statistics

To test the differences between varieties and N supplies, data were analyzed using two-way analysis of variance (ANOVA) and the least significant difference (LSD) test with the SAS 9.0 statistical software package. Different lowercase letters were used to indicate significant differences (*P*<5%) among N supplies, and different uppercase letters were used between varieties.
